# Cerebro-Spinal Fever

**Published:** 1875-03

**Authors:** 


Cerebro-Spinal Fever.—Dr. J. B. Hamilton, U. S. Army, reports (*New York Medical Journal*, February, 1875,) the details of eleven cases of cerebro-spinal meningitis, of a very severe type, among those occurring in his private practice in Illinois in the winter of 1873–4. He does not believe that it is an inflammation, but regards it as a zymotic disease, with the fever and localized inflammation dependent upon miasmatic poison. He asserts that: "In the beginning the skin is always dry; the secretions throughout the body are arrested; * * the patient often dies from retention in the blood of excrementitious material. When the excreting organs are at once overpowered by the malarial poison introduced from without, the patient dies suddenly, a victim to chemical forces generated within his own body. No treatment will be of any avail which does not have *elimination* for its primary object." So far as general treatment can be outlined he recommends the following: For the first day or two, ℞. Ext. ergot. fl. f℥j., sp. ammon. arom., f℥ij. A teaspoonful in a little water every four hours. ℞. Potass. acetat., ℨxij., aquæ camph., f℥vj. A tablespoonful every two hours until diuresis is produced. Additionally he uses the warm bath followed by wrapping in flannel or rubbing with dry mustard every three, four or six hours, and employs stimulants, if necessary, from the outset. As the symptoms ameliorate the ergot mixture may be diminished in frequency, and at the third or fourth day discontinued. Then large doses of quinine may be useful and a more stimulating diuretic may be substituted. In his cases the sequelæ responded best to potassic iodide.

